# Association Medical Officers Confederate States Army and Navy

**Published:** 1875-02

**Authors:** 


### Editorial.

---

We are informed that the meeting of the Association of Medical Officers of the Confederate States Army and Navy, which adjourned from Atlanta to re-convene in Richmond in July next, has been postponed to the 19th October following. The medical Association of Virginia holds its annual session on the succeeding day, the 20th October.

We have received from the Smithsonian Institution at Washington the following queries:

1. To what extent do you see chorea (of childhood) among whites?

2. Is it found more in one locality than another?

3. At what season do the attacks come?

4. How often have you seen chorea in blacks of pure breed? If possible, give cases.

5. How common is it in mulattos?

Any of our readers who can furnish the above information, directed to Prof. Joseph Henry, at Washington, will help to advance medical science, and receive proper credit for their contributions.

